# Optical Acceleration Measurement Method with Large Non-ambiguity Range and High Resolution via Synthetic Wavelength and Single Wavelength Superheterodyne Interferometry

**DOI:** 10.3390/s18103417

**Published:** 2018-10-12

**Authors:** Qianbo Lu, Dexin Pan, Jian Bai, Kaiwei Wang

**Affiliations:** State Key Laboratory of Modern Optical Instrumentation, Zhejiang University, Hangzhou 310027, China; lqb@zju.edu.cn (Q.L.); pdx@zju.edu.cn (D.P.); bai@zju.edu.cn (J.B.)

**Keywords:** acceleration measurement, synthetic wavelength, superheterodyne, large non-ambiguity range, high resolution

## Abstract

Interferometric optomechanical accelerometers provide superior resolution, but the application is limited due to the non-ambiguity range that is always less than half of the wavelength, which corresponds to the order of mg. This paper proposes a novel acceleration measurement method based on synthetic wavelength and single wavelength superheterodyne interferometry to address this issue. Two acousto-optical modulators and several polarizers are introduced to the two-wavelength interferometry to create four beams with different frequencies and polarization states, and two ultra-narrow bandwidth filters are used to realize the single wavelength measurement simultaneously. This technique offers the possibility to expand the non-ambiguity range without compromising the high resolution. Also, the superheterodyne phase measurement and the corresponding processing algorithm are given to enable real-time measurement. A prototype is built and the preliminary experimental results are compared with the simulation results, showing good agreement. The results prove an estimated acceleration measurement resolution of around 10 μg and a non-ambiguity range of larger than 200 mg, which is more than 100 times that of the single wavelength-based optical accelerometer.

## 1. Introduction

The measurement of acceleration is necessary for a variety of applications ranging from consumer electronics to military equipment [1,2]. For example, inertial navigation and gravitational wave detection pose a significant challenge at an extremely high resolution, typically on the order of micro-g to nano-g [3,4]. This requires a low noise level and superior sensitivity, which involve the acceleration-displacement sensitivity of a micromachined structure [5,6,7] and displacement measurement sensitivity [8,9,10,11]. Optomechanical accelerometers based on interferometric readouts are capable of meeting the requirement of high resolution because of the high displacement resolution achieved by optical methods and good electromagnetic immunity [12,13,14,15,16]. The interferometric readout measures the intensity of output light, which is related to the displacement of a proof mass. This displacement has a certain relationship with the applied acceleration. Although the forms of interferometric readouts can be different, the relationships between the acceleration and the output light intensity are similar, as in [9]:

(1) $$I(a)=I_{b}+AI_{0}\sin^{2}(\frac{4\pi a}{\lambda }),\;$$

where *a* is the applied acceleration, *I_b_* is the bias intensity, *I*_0_ is the initial intensity, *A* is the scale factor, and *λ* is the wavelength of the laser. Because the measurement result is interpreted by the phase that uses the wavelength as a ruler, there is a trade-off between the sensitivity and the non-ambiguity range (NAR). Conventional high-resolution interferometric accelerometers are mainly based on single wavelength schemes. It has been confirmed that a single wavelength optomechanical accelerometer can achieve a noise equivalent acceleration of tens of ng/√Hz and a resolution of μg at a low-frequency regime combined with a highly sensitive mechanical structure [14,17]. However, the NAR is limited to a half- or even quarter-wavelength, always less than 1 μm, which equals a tiny acceleration. To take Loh’s device as an illustration [14], this device can achieve a sensitivity of around 10,000 V/g and a noise level of 0.1 μg/√Hz, but the NAR corresponds to an acceleration range of 4.06 mg according to the acceleration-displacement sensitivity of 39 μm/g, and the linear region is smaller than this value. Although a continuous measurement can be carried out with a fast update rate to determine the absolute acceleration without loss of movement, the update rate should be incredibly high for dynamic acceleration even with a small amplitude. In our previous work [15], we tested periodic acceleration with an amplitude of 5 mg at 5 Hz. For this extremely small acceleration and low frequency, the sampling rate should be 30 kHz in order to keep up with the acceleration.

As for the displacement measurement, there have been plenty of demonstrations of overcoming the NAR issue, including two-wavelength schemes [18,19,20], multi-wavelength schemes [21], frequency comb schemes [22,23], wavelength scanning schemes [24], etc. By using a larger synthetic wavelength, the NAR can be expanded by several orders of magnitude. Nevertheless, there is presently no interferometric accelerometer that employs the aforementioned techniques to resolve the conflict between sensitivity and NAR. The main reason for this is that interferometric accelerometers now stop at the stage of principle specimen machines; thus, the NAR issue is not the great concern. Although there are some force-feedback designs which enable altering device dynamics in situ, this poses a significant challenge to the high precision force control and can even downgrade the performance compared with the open-loop schemes [17,25]; thus, optical accelerometers suffer from phase ambiguity, and their NAR are constrained to less than half a wavelength, which corresponds to acceleration on the mg order [26,27]. In addition, real-time measurement and high precision are required, because the acceleration measurement is usually an on-line measurement, which has to measure dynamic accelerations in different areas.

Inspired by these developed optical measurement techniques, we first demonstrate a real-time large NAR and high-resolution optical acceleration measurement approach that makes use of the merits of synthetic wavelength and singe wavelength interferometry along with the superheterodyne detection method [28]. This new scheme offers the possibility of acceleration measurement with a large NAR in real time without compromising the resolution. A prototype is built and tested in which we implement multi-wavelength interferometry by generating four different frequencies with different polarization states. An ultra-narrow bandwidth optical filter and a specific signal processing method are introduced to realize the high-resolution single wavelength and the large NAR synthetic wavelength measurements simultaneously. The acceleration is determined by the superheterodyne phase detection scheme to enable real-time measurement. Preliminary experimental results are obtained and compared with the simulation results, which show good agreement. The estimated resolution is on the μg level and the NAR is increased by two orders of magnitude compared with the single wavelength-based scheme, which proves our scheme as a potential candidate for large NAR and high-resolution acceleration measurement.

## 2. Optical Acceleration Measurement Principle

**Figure 1:**
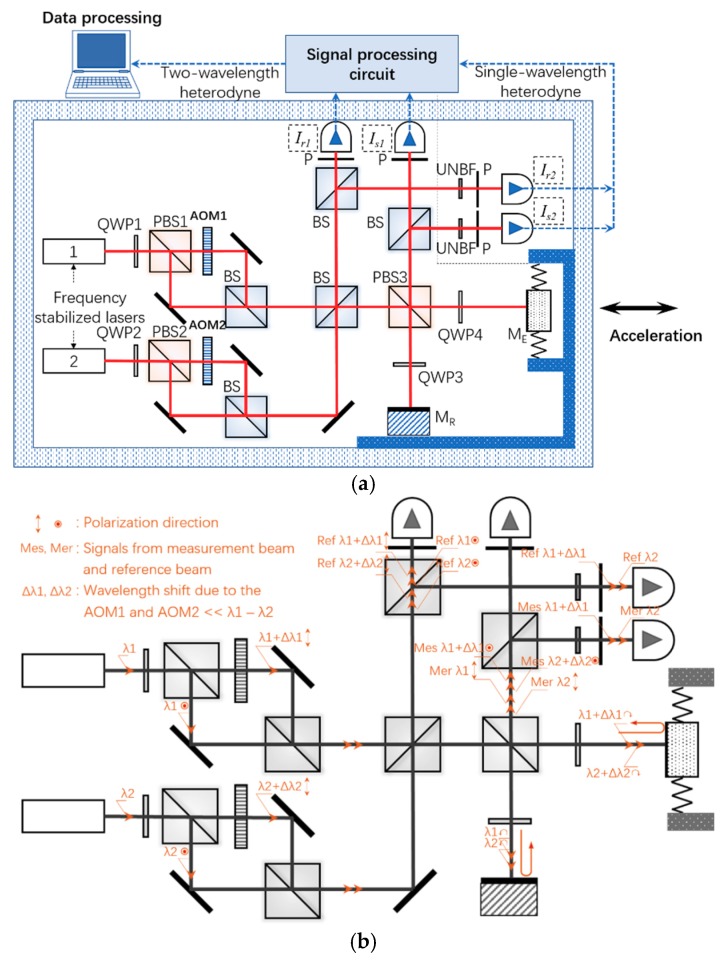
(**a**) Schematic diagram of optical acceleration measurement method based on synthetic wavelength and single wavelength superheterodyne detection. QWP, quarter-wave plate; PBS, polarization splitting prism; AOM, acousto-optical modulator; BS, beam splitter; P, polarizer; UNBF, ultra-narrow bandwidth filter; M_R_, micro mirror; M_E_, micromachined elastic structure. (**b**) Optical layout of the configuration with marks of polarization direction and wavelength of the laser beams.

Figure 1a shows the overall configuration of the optical acceleration measurement approach with a large NAR and high resolution. Two frequency-stabilized (single longitude mode) lasers are adopted to serve as the wavelength ruler, with wavelengths *λ*_1_ and *λ*_2_, respectively, and *ν*_1_ = *c*/*λ*_1_ and *ν*_2_ = *c*/*λ*_2_ are the corresponding light frequencies of these two laser sources, while *c* is the speed of light in air. *λ_s_* = *λ*_1_*λ*_2_/(*λ*_1_ − *λ*_2_) is the synthetic wavelength. Each laser beam from the sources passes through a quarter-wave plate (QWP) and a polarization splitting prism (PBS) to create two orthogonal polarized beams with equal light intensity. Two acousto-optical modulators (AOMs) are introduced to create two frequency shifts, where the frequency differences are *f*_1_ and *f*_2_, respectively. The frequency shifts introduced by the AOMs are usually on the order of tens of MHz, which are orders of magnitude smaller than *ν*_1_ and *ν*_2_; hence, the wavelength shift Δ*λ*_1_ = *f*_1_*λ*_1_^2^/(*c* + *f*_1_*λ*_1_) ≈ *f*_1_*λ*_1_/*ν*_1_ and Δ*λ*_2_ = *f*_2_*λ*_2_^2^/(*c* + *f*_2_*λ*_2_) ≈ *f*_2_*λ*_2_/*ν*_2_ due to the frequency shifts can be easily calculated to be much smaller than the original wavelengths and the wavelength difference *λ*_1_ − *λ*_2_. Then, these four lasers illuminate an extended form of Mechelson-type superheterodyne interferometer. A fixed M_R_ and a M_E_ normal to the laser direction constitute two arms of the interferometer. Two large-bandwidth photodetectors along with appropriate polarizers are implemented to produce the synthetic wavelength reference and measurement signals. Notably, the polarization axis of the polarizers has an angle of 45° between two perpendicular polarization directions, the same as the polarizers behind two UNBFs. In order to maintain a high resolution simultaneously, the reference and measurement signals are split so that a portion of the light can be used to achieve the single wavelength heterodyne measurement. Two ultra-narrow bandwidth filters (UNBFs) are adopted to extract the signals with wavelengths of *λ*_1_ and *λ*_1_ + Δ*λ*_1_, because the ultra-small wavelength shift can be neglected for the wavelength window of the filter.

The light path, including the polarization direction and wavelength marks, is performed in Figure 1b. The laser beams are distinguished by different frequencies (wavelengths) as well as the polarization directions. Δ*f* = *f*_1_ − *f*_2_ is the superheterodyne frequency that allows direct measurement of the phase change via the ruler of the synthetic wavelength, and the different polarization directions help to avoid negative impacts from the reflected laser.

The synthetic wavelength reference intensity signal *I_r_*_1_ and the synthetic wavelength measurement intensity signal *I_s_*_1_ have the following forms:

(2) $$I_{r1}=4A_{0}^{2}+2A_{0}^{2}\left[\cos(2\pi f_{1}t)+\cos(2\pi f_{2}t)\right],\;$$

(3) $$I_{s1}=4A_{0}^{2}+2A_{0}^{2}\left[\begin{array}{l} \cos(2\pi f_{1}t+\frac{4\pi Sa}{\lambda_{1}}-\varphi_{1})\\ +\cos(2\pi f_{2}t+\frac{4\pi Sa}{\lambda_{2}}-\varphi_{2}) \end{array}\right],\;$$

where *A*_0_ is the amplitude of each frequency laser beam, *S* denotes the acceleration-displacement sensitivity of the elastic sensing structure, *a* represents the applied acceleration, $\varphi_{1}$ and $\varphi_{2}$ are the initial phases of two interference arms, which can be described as $2\pi z_{1}/\left(\lambda_{1}+\Delta \lambda_{1}\right)$ and $2\pi z_{2}/\left(\lambda_{2}+\Delta \lambda_{2}\right)$, and *z*_1_ and *z*_2_ denote the initial displacements. Each signal is obtained and fed to a designated heterodyne phase-detection circuit, which contains two amplifiers, two electronic mixers, and bandpass filters as well as a phase meter. After passing through the bandpass filter, the synthetic wavelength reference and measurement signals are described as

(4) $$R_{1}=A_{01}\cos\left[2\pi (f_{1}-f_{2})t+\varphi_{01}\right],\;$$

(5) $$M_{1}=A_{02}\cos\left[2\pi (f_{1}-f_{2})t+\varphi_{02}+\frac{4\pi Sa}{\lambda_{s}}\right],\;$$

where *A*_01_ and *A*_02_ are the scaling factors, and $\varphi_{01}$ and $\varphi_{02}$ are the initial phases of the processed reference and measurement signals, respectively. By detecting the phase change 4*πSa*/*λ_s_*, we can obtain the acceleration via the ruler of the synthetic wavelength *λ_s_*.

The single frequency reference and measurement signals obtained by two detectors can be expressed as

(6) $$I_{r2}=2A_{0}^{2}+A_{0}^{2}\cos(2\pi f_{1}t),\;$$

(7) $$I_{s2}=2A_{0}^{2}+A_{0}^{2}\cos(2\pi f_{1}t+\frac{4\pi Sa}{\lambda_{1}}-\varphi_{1}).\;$$

After passing through the heterodyne phase detection circuit, the phase difference 4*πSa*/*λ*_1_ can be obtained via the ruler of the single wavelength *λ*_1_. 

**Figure 2:**
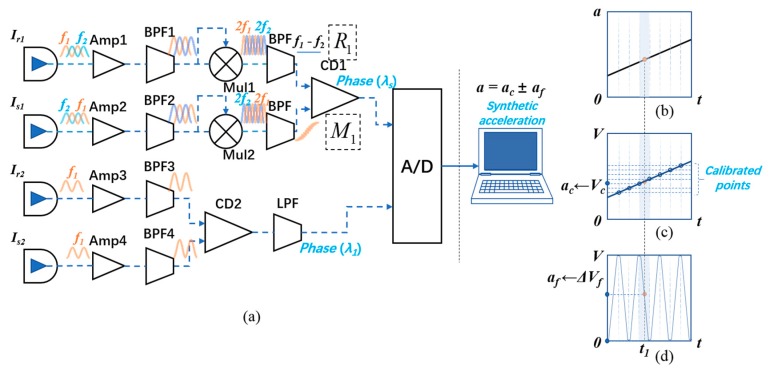
(**a**) Sketch of the signal processing circuit, including amplifiers, band pass filters, self-mixers, and coherent-demodulators. (**b**) Original linear acceleration as a function of time. (**c**) Phase output via the ruler of the synthetic wavelength as a function of time. (**d**) Phase output via the ruler of the single wavelength as a function of time.

The signal process scheme is presented in Figure 2a and is composed of a synthetic wavelength superheterodyne phase detection circuit and a single wavelength heterodyne interference phase detection circuit. The detected phases via the rulers of *λ_s_* and *λ*_1_ are combined to get the actual acceleration by a specific algorithm after A/D conversion.

As for the synthetic wavelength superheterodyne process, the optical intensities expressed by Equations (2) and (3), which contain *f*_1_ and *f*_2_ frequency components, are converted into voltage signals first, along with AC amplification. After shaping filtering by amplifiers (Amps) and band pass filters (BPFs), the signals pass through the self-mixers (Mul) to obtain difference frequency signals and multiple frequency signals. The difference frequency signals are extracted by BPFs. The phase of the reference signal is a constant, whereas the phase of the measurement signal carries the acceleration information via the ruler of the synthetic wavelength *λ_s_*. In general, the acceleration, or the phase change, has a relatively lower frequency compared with the frequency difference Δ*f*, which is usually on the order of tens of kHz. Therefore, we can obtain the coarse phase via a coherent-demodulator (CD1). For the single wavelength heterodyne process, the reference and measurement signals are input into another coherent-demodulator (CD2), which operates at a higher frequency regime. The fine phase that contains the information of acceleration can be extracted via the ruler of the single wavelength *λ*_1_. The output of the coherent-demodulators, or phase detectors, is a triangle wave for a linear phase variation, which means the voltage is proportional to the phase when the phase is smaller than *π*, while it has a negative correlation with the phase when the phase is between *π* and 2*π*. 

The voltage signals that carry the fine phase and coarse phase are converted into digital signals, and then are processed by a specific algorithm. As shown in Figure 2b–d, the coarse acceleration obtained by the coarse phase (termed as *a_c_*) and the fine acceleration obtained by the fine phase (termed as *a_f_*) are combined to synthesize the exact acceleration. For variable acceleration, the acceleration at a certain time, *t*_1_, corresponds to two values of acceleration via two different rulers when four signals are synchronously sampling. In practice testing conditions, the response of the coarse ruler can be calibrated via a higher-resolution detection method (for example, a high-precision accelerometer based on single wavelength scheme), dividing the response of the synthetic wavelength into a lot of parts. To calculate the final acceleration at a certain time, we add these two accelerations, which can be described as *a* = *a_c_* ± *a_f_*. Here, *a_c_* is the acceleration that corresponds to the lower limit value in the interval of the response of the synthetic wavelength at *t*_1_, and *a_f_* is the acceleration variation corresponding to the variation of the output in this interval of the response of the single wavelength from the start point to *t*_1_. The plus or minus depends on the slope of the actual acceleration: plus corresponds to the case that the slope is positive and minus corresponds to the case that the slope is negative. The slope can be approximated by that of the response of the synthetic wavelength if the synthetic wavelength is large enough. It is worth mentioning that if the interval is larger than one period of the response of the single wavelength, the number of the period of the single wavelength should be counted from the beginning of the interval. This can be avoided by precise calibration of the synthetic wavelength response so that the interval spacing is small enough. However, the number of the interval should not exceed *range/noise level_s_*, where *range* denotes the output range of the synthetic wavelength and *noise level_s_* is the noise level of the output.

**Figure 3:**
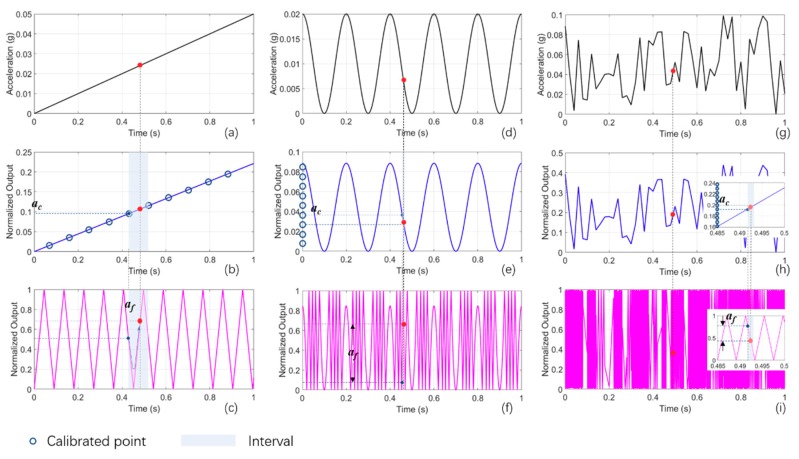
Simulation results for three types of acceleration. (**a**) Acceleration varies with time linearly from 0 to 1 s, and amplitude from 0 to 0.05 g. (**b**) Normalized phase ouput of synthetic wavelength for linear acceleration. (**c**) Normalized phase output of single wavelength for linear acceleration. (**d**) Acceleration with a cosine relationship of time; amplitude is 0.01 g. (**e**) Normalized phase output of synthetic wavelength for cosinoidal acceleration. (**f**) Normalized phase output of single wavelength for cosinoidal acceleration. (**g**) Stochastically varying acceleration as a function of time. (**h**) Normalized phase output of synthetic wavelength for random acceleration; inset: enlarged view of response from 0.485 to 0.5 s. (**i**) Normalized phase output of single wavelength for random acceleration; inset: enlarged view of response from 0.485 to 0.5 s.

In order to better understand the process, we simulate some cases of the acceleration response. First, we set the acceleration to vary with time linearly, as shown in Figure 3a. The acceleration changes from 0 g to 0.05 g in 1 s, the acceleration-displacement sensitivity *S* is set to 70 μm/g [29], the single wavelength is 632.8 nm, and the synthetic wavelength is 100 times the single wavelength according to our experimental setup. The normalized voltage response as a function of time via the ruler of the synthetic wavelength and the single wavelength are depicted by Figure 3b,c, respectively. It is evident that the synthetic wavelength response tracks the variable acceleration well, which means the acceleration change can be approximated by the voltage variation of the synthetic wavelength response linearly, whereas the response of the single wavelength has a lot of periods. Once the coarse acceleration detection via the synthetic wavelength is well calibrated, the output of the synthetic wavelength signal is separated into several intervals, denoted by the pink-square regions; an interval is defined by two precise calibrated points, presented by the purple circles. The coarse acceleration *a_c_* is read from the discrete point (Figure 3b), and the fine acceleration *a_f_* is obtained through the variation of the output of the single wavelength (Figure 3c). The final acceleration is calculated by *a* = *a_c_* + *a_f_* because of the positive slope of the synthetic wavelength response in this interval. Likewise, a cosinoidal acceleration and a random acceleration at a certain time, as shown in Figure 3d,g, respectively, can also be obtained through the calibrated points of the outputs of the synthetic wavelength (Figure 3e,h) and the variations of the outputs of the single wavelength in the interval, as shown in Figure 3f,i. The final accelerations are calculated by *a* = *a_c_* − *a_f_* for the cosinoidal acceleration because of the negative slope of the response of the synthetic wavelength in the interval, while *a* = *a_c_* + *a_f_* for the random acceleration due to the positive slope. The only difference is that the responses of the single wavelength are no longer periodic. To summarise, combining two-wavelength-based rulers as similar as the vernier caliper, this scheme enables a high-resolution acceleration measurement without compromising the NAR. 

## 3. Prototype Setup and Experimental Results

**Figure 4:**
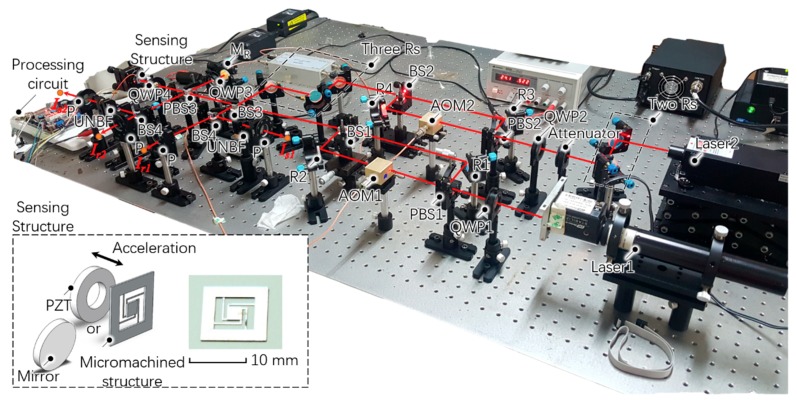
Prototype for the optical acceleration measurement configured in this investigation. Inset: sketch of the sensing structure, which contains a ring piezoelectric transducer and a micromachined structure or a mirror.

A prototype was built to verify the feasibility of this measurement technique, as shown in Figure 4. The wavelengths of laser 1 (Model 05-STP-912, Melles Griot Inc., Carlsbad, CA, USA) and laser 2 (Model MSL-FN-639-S, Changchun New Industries Optoelectronics Tech. Inc., Changchun, China) were *λ*_1_ = 632.8 nm and *λ*_2_ = 639 nm, respectively, hence the synthetic wavelength was 65.22 μm; the frequency shifts introduced by two AOMs (Qingjin Optoelectronics Tech. Inc., Shanghai, China) were *f*_1_ = 100.00 MHz and *f*_2_ = 100.05 MHz, and the frequency difference was 50 kHz, which was the heterodyne frequency; the sensing structure should be a crab-shaped cantilever-mass structure, shown in the inset of Figure 4; however, herein we replaced it by a small reflectance mirror because the prototype was not integrated; a ring piezoelectric transducer (PZT; CMBR03 Noliac Inc., Kvistgaard, Denmark) was used to apply acceleration to the sensing structure; two ultra-narrow bandwidth optical filters (Model FL05632.8-1, Thorlabs Inc., Newton, NJ, USA) with a center wavelength of 632.8 nm and full width at half maximum of 1 nm were implemented to extract the single wavelength signals. There are some slight differences between the setup and the schematic diagram. Several additional mirrors, such as “two Rs” and “three Rs”, were used to offer adjustment over multiple degrees of freedom to the laser beams; an attenuator was introduced to adjust the relative intensity of these two incident laser beams so that we could obtain a high contrast. Because the output *I_r_*_1_ and *I_r_*_2_ have two frequencies of around 100 MHz, two photodiodes (Model PDA10A, Thorlabs Inc., Newton, NJ, USA) with large bandwidth (larger than 300 MHz) were employed to obtain the reference signals. AD8302 was used to obtain the phase outputs of both the synthetic and single wavelength signals, whose noise level was 10 mV/degree and output range was 0 to 1.8 V.

**Figure 5:**
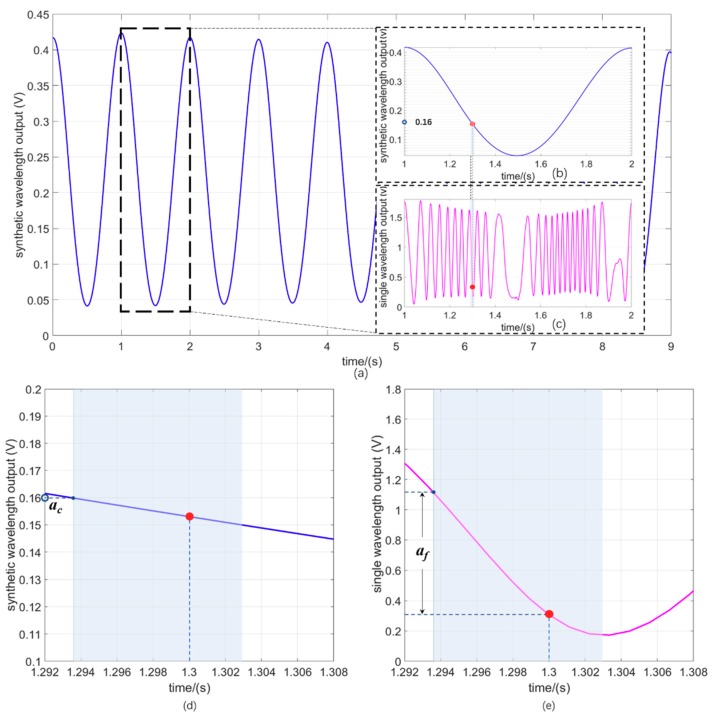
Measurement results of (**a**) synthetic wavelength output versus sampling time in 9 s; (**b**) synthetic wavelength output versus sampling time from 1 to 2 s along with calibrated intervals; (**c**) corresponding single wavelength output versus sampling time from 1 to 2 s. (**d**) Enlarged view of synthetic wave output versus sampling time from 1.292 to 1.308 s; pink area represents an interval where the specific time 1.3 s is located. (**e**) Enlarged view of single wave output versus sampling time from 1.292 to 1.308 s.

A sinusoidal driving voltage was applied to the PZT, which had an amplitude of 200 V and a frequency of 1 Hz. Combined with the responsivity of the PZT, which was 15 nm/V, the corresponding displacement denoted by *Sa* in Equations (5) and (7) was calculated to be 3 μm at a frequency of 1 Hz. The phase responses of the synthetic wavelength and the single wavelength are presented in Figure 5. Figure 5a depicts the synthetic wavelength output versus the sampling time in a relatively long period, and Figure 5b,c represent the enlarged view of the synthetic wavelength output and the corresponding single wavelength output in 1 s. The output of the AD8302 is a voltage as a function of the phase, which has a responsivity of 573 mV/radians when the phase difference is in the range of −*π* + 2n*π* to 0 + 2n*π*, where n is an integer, and a responsivity of −573 mV/radians when the phase difference is in the range of 0 + 2n*π* to *π* + 2n*π*. We can finally read the displacement (or the acceleration) at a specific time. Combining the acceleration-displacement sensitivity of the sensing structure, which is 76.06 μm/g [15,29], the NAR of the synthetic wavelength response is calculated to be around 0.214 g, more than 100 times larger than the original NAR of the single wavelength-based scheme, which was 2.08 mg. Hence the response of the synthetic wavelength can easily be calibrated by using an accelerometer with an accuracy of 1 mg, dividing the output into 180 intervals, with each interval corresponding to 10 mV (1.256 mg).

For the measured acceleration at 1.3 s, it is found that the synthetic wavelength output lies within the range of 0.15 to 0.16 V. We therefore obtain the *a_c_* from the lower limit of the interval, which is marked in Figure 5d, and the value is 0.16 V, corresponding to an acceleration of 19 mg (*Sa* is 1.449 μm). The fine acceleration *a_f_* is obtained by the difference between the start point and the point at 1.3 s in the interval of the single wavelength output, which is 0.784 V, corresponding to 0.9059 mg (*Sa* is 68.9 nm). The accuracy mainly depends on the phase measurement accuracy of the single wavelength measurement. According to the previous work, the noise level of the whole system except the phase meter was around 1 mV [11,28], wihch is much smaller than the noise level of the AD8302 (~10 mV); therefore, we get the estimated resolution of this prototype as

(8) $$\mathrm{resolution}=\frac{\mathrm{noise}\;\mathrm{level}}{\mathrm{sensitivity}}=\frac{10\;\mathrm{mV}}{1800\;\mathrm{mV}}\times \frac{0.6328\;\mu m}{4\times 76.06\;\mu m/g}=11.5\;\mu g.\;$$

**Figure 6:**
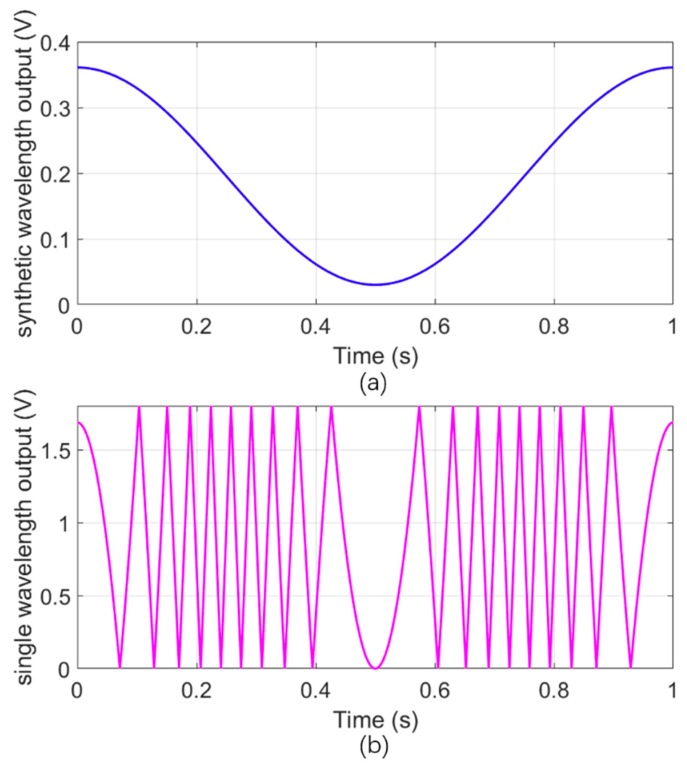
(**a**) Simulated synthetic wavelength output for the cosinoidal acceleration (displacement) in 1 s, in which the synthetic wavelength is set to 65.22 μm, and the acceleration-displacement sensitivity is 76.06 μm/g. (**b**) Simulated single wavelength output for the cosinoidal acceleration (displacement) in 1 s, in which the single wavelength is 632.8 nm.

The final acceleration at 1.3 s can be calculated as *a_c_* − *a_f_* = 19 mg − 0.9059 mg = 18.0941 mg. We also give the simulated response of both the synthetic wavelength and the single wavelength considering the actual parameters, as shown in Figure 6a,b. Figure 5b,c coincide well with the simulated results: the differences contain the period number of the simulation result of the synthetic wavelength in 1 s is 10, while the period number of the experimental result is 11; the amplitude of the experimental result varies with time and is slightly different from the ideal value. The former deviation could be due to the experimental errors involved in the alignment and the nonlinearity of the PZT, whereas the latter one could be due to the phase measurement and the data processing circuit errors.

Notably, the resolution and NAR of the acceleration measurement approach can be readily adjusted by changing the sensing structure and the wavelength difference between two laser sources, which are related to the acceleration-displacement sensitivity and the synthetic wavelength, respectively. The accuracy can be further improved by using a phase meter and a processing circuit with a higher phase measurement accuracy because it is the major limitation. 

## 4. Conclusions

In this paper, an optical acceleration measurement method based on synthetic wavelength and single wavelength superheterodyne detection is presented to achieve a large NAR without compromising the single wavelength-based high resolution. The measurement principle and the scheme design are provided. A prototype is established to verify the validity and feasibility of this method. The experimental results coincide well with the simulation results and demonstrate that this method can realize a resolution of around 10 μg and an NAR of 214 mg, which improves the NAR by more than two orders of magnitude without loss of resolution. Moreover, the NAR can be further expanded by using a smaller wavelength difference for two laser sources, and the resolution can be tuned by using a high-precision phase measurement approach. This technique holds promise for integration [30] and offers the possibility to resolve the conflict between NAR and resolution, thus allowing the optical accelerometers to be applied to a wide range of applications.
